# Wessen Perspektiven zählen? Leerstellen und Anforderungen für eine nachhaltige Beteiligung junger Menschen in der Politikberatung

**DOI:** 10.1007/s12054-022-00541-9

**Published:** 2022-12-06

**Authors:** Walburga Hirschbeck

**Affiliations:** Deutsches Jugendinstitut e.V., München, Deutschland

**Keywords:** Beteiligung, Politikberatung, Junge Menschen, Generationengerechtigkeit, Sozial Ungleichheit, Politische Bildung

## Abstract

Das Recht auf Beteiligung junger Menschen wird im Kontext der Politikberatung noch nicht ressortübergreifend und strukturell umgesetzt. Bisherige Formate weisen oftmals einen projekthaften Charakter auf und sind intransparent in Hinblick auf die tatsächlichen Mitgestaltungsmöglichkeiten junger Menschen. Eine inklusive und nachhaltige Beteiligung setzt die Bereitschaft voraus, Entscheidungsmacht zu teilen, gezielt Zugänge für benachteiligte junge Menschen zu entwickeln und Beteiligung mit Bildung zu verbinden.

Eine ernst gemeinte und nachhaltig verankerte Beteiligung der jungen Generation in der Politikberatung geht über das bloße Befragen und Anhören von Anliegen hinaus. Vor dem Hintergrund der ungleichen Machtverhältnisse im generationalen Gefüge umfasst Beteiligung auch, jungen Menschen Wirkmacht zu überlassen. Um mehr Chancengleichheit bei der Beteiligung junger Menschen zu erreichen, kommt dem breiten und niedrigschwelligen Zugang zu Beteiligungsangeboten eine bedeutende Rolle zu.

Auf verschiedenen Ebenen hat sich ein Bewusstsein dafür entwickelt, junge Menschen mehr an politischen Prozessen zu beteiligen und sie als Grundrechtsträger_innen stärker in die politischen Aushandlungs- und Entscheidungsprozesse einzubeziehen, anstatt sie nur anwaltschaftlich zu vertreten.

## Zwischen ebenenübergreifendem Paradigmenwechsel und kleinen (Rück‑) Schritten

In zahlreichen Kommunen gibt es mittlerweile Kinder- und Jugendparlamente, Jugendgemeindebeiräte oder Jugendbeiräte, die in unterschiedlichem Ausmaß an kommunalen Entscheidungsprozessen beteiligt sind. Auf Bundesebene wurde Kinder- und Jugendbeteiligung in der Politikberatung insbesondere durch die Jugendstrategie der Bundesregierung realisiert, die sich seit 2019 in der Umsetzungsphase befindet. Im Rahmen der Jugendstrategie konnten junge Menschen an Veranstaltungen und Projekten wie Jugend-Audits, Jugend-Politiktage, dem Jugend-Budget oder einem Planathon teilnehmen, die zum Ziel haben, die Perspektiven junger Menschen zu erfragen und diese einzubeziehen. Dabei handelt es sich um die Ausrichtung teilweise regelmäßiger, jedoch punktueller konsultativer Formate.

Eine der wenigen strukturell verankerten Beteiligungsformen auf Bundesebene hat das Bundesministerium für wirtschaftliche Zusammenarbeit und Entwicklung (BMZ) eingerichtet. Dort wurde im Mai 2021 ein Jugendbeirat eingesetzt, der das BMZ beraten, sich in die Entwicklung übergreifender Strategien und Konzepte einbringen, Vorschläge (für weitere Jugendbeteiligung) unterbreiten sowie Positionspapiere verfassen kann (GIZ 2022). Politikberatung durch junge Menschen auf europäischer Ebene findet im Rahmen der zweiten EU-Jugendstrategie statt und konzentriert sich auf die drei Kernbereiche Beteiligung, Begegnung und Befähigung. Jugendbeteiligung in Europa soll vor allem durch die neue Programmgeneration von Erasmus+ über neu eingeführte Jugendpartizipationsprojekte gefördert werden. Diese umfassen unter anderem Veranstaltungen wie Workshops, Treffen, Seminare und Konsultationen. Die unterschiedlichen Beispiele zeigen, dass auf allen Ebenen, von der Kommune bis zur EU, eine Bereitschaft erkennbar ist, junge Menschen zu beteiligen.

Gleichzeitig hat spätestens die Covid-19-Pandemie die fehlende Krisenfestigkeit der Beteiligung junger Menschen auf allen Ebenen offenbart. Junge Menschen sahen ihre Anliegen und Bedarfe in politischen Entscheidungen nicht vertreten und wurden nicht oder sehr spät um ihre Einschätzung gefragt (Andresen et al. 2020a, b, 2022). Kinder- und Jugendbeteiligung im Kontext der Politikberatung auf kommunaler, Landes‑, Bundes- und EU-Ebene ist bisher noch weit davon entfernt, eine krisensichere Selbstverständlichkeit zu sein und bleibt hinter den Vorgaben der UN-Kinderrechtskonvention zurück (Nadjafi-Bösch 2022). Anstatt eine durchgängige Beteiligung strukturell zu verankern und jungen Menschen die Möglichkeiten zu geben, die zu beratenden Themen selbst zu bestimmen, werden sie noch zu oft maximal aufgefordert, auf Themenstellungen von Erwachsenen zu reagieren. Für Veranstaltungen über oder mit der Beteiligung junger Menschen gibt es oft keine Mindestanforderungen an die Partizipation.

## Projekthafter Charakter und Gefälle in der ressortübergreifenden Beteiligung

Bis auf der kommunalen Ebene fehlt eine ressortübergreifende langfristig strukturell verankerte Beteiligung von Kindern und Jugendlichen an politischen Entscheidungsprozessen. Die junge Generation erfährt jedoch Politik nicht nur in ihrem Sozialraum, sondern ebenso auf Bundes- und EU-Ebene beispielsweise in der Wohnungsbaupolitik, der Migrationspolitik oder der Arbeitsmarktpolitik. Eine ressortübergreifende Beteiligung junger Menschen an bundespolitischen Beratungsprozessen ist trotz einer gemeinsamen Jugendstrategie der Bundesregierung nicht erkennbar. Die meisten Beteiligungsangebote auf Bundesebene finden ohne strukturelle Verstetigung im Rahmen punktueller Veranstaltungen statt, deren Ernstcharakter nicht immer offensichtlich ist. Bisherige Beteiligungsprojekte konzentrieren sich auf wenige Ministerien. Während insbesondere das BMFSFJ, das BMU sowie das BMZ vermehrt Anstrengungen unternehmen, die Ziele der Jugendstrategie umzusetzen, sind andere Ministerien wie das BMF, das BMWK oder das BMVG möglicherweise gerade dabei, sich leise Gedanken über Kinder- und Jugendbeteiligung zu machen, obwohl beispielsweise das Bundesministerium der Finanzen durch weitreichende Entscheidungen die Entwicklungsmöglichkeiten der jungen Generation massiv beeinflusst. Bei punktuellen Veranstaltungen werden in der Regel der Rahmen und oftmals auch die diskutierten Themen von den erwachsenen Akteuren bestimmt. Der projekthafte Charakter verhindert eine strukturell angelegte Beteiligung, die notwendig wäre, damit junge Menschen permanent für sie relevante Themen einbringen und damit sie sich auch bei langwierigen politischen Prozessen, beispielsweise der bevorstehenden Kindergrundsicherung oder bei Änderungen in der Wohnungsbaupolitik, durchgehend beteiligen können.

Wenngleich eine Öffnung in der Diskussion um Kinder- und Jugendbeteiligung in der Politikberatung zu beobachten ist, werden junge Menschen dennoch nicht systematisch ressortübergreifend beteiligt. Trotz aller Bekenntnisse zur hohen Relevanz von Beteiligung können junge Menschen bisher vorwiegend auf einer von Erwachsenen festgelegten Spielwiese ihre Anliegen kundtun.

## Die Legende von der Augenhöhe

*„Ich finde politisches Engagement von Schülerinnen und Schülern toll. Von Kindern und Jugendlichen kann man aber nicht erwarten, dass sie bereits alle globalen Zusammenhänge, das technisch Sinnvolle und das ökonomisch Machbare sehen. Das ist eine Sache für Profis. CL“ *(Christian Lindner, twitter, 10.03.2019) Während sich seit dem Zitat zum Engagement junger Menschen im Rahmen von Fridays for Future vor über drei Jahren auf mehreren Ebenen bei der Beteiligung junger Menschen viel bewegt und das Bewusstsein für das Recht junger Menschen auf Beteiligung verstärkt hat, ist zur gleichen Zeit der Autor dieses Zitats Bundesfinanzminister geworden. Hinter den Worten erscheint (mir) eine Haltung, die Kompetenzen und Erfahrungen junger Menschen abwertet und sie delegitimiert, sich an Entscheidungsprozessen beteiligen zu können. Obwohl das Recht auf Beteiligung in Art. 12 UN-KRK kein umfassendes Verständnis aller globalen Zusammenhänge voraussetzt, hält sich nicht nur im politischen Kontext die Einschätzung, dass nachhaltige Entscheidungen nur von sogenannten Profis – erwachsenen Menschen (mit einem hohen Bildungsabschluss) – getroffen werden können.

Solange junge Menschen bloß im Rahmen von feststehenden (Alibi)veranstaltungen ihre Anliegen vortragen können, die Informationen jedoch nicht in politische Entscheidungsprozessen miteinfließen, ist die Beteiligung junger Menschen in der Politikberatung auf Bundesebene auf das gnädige Gewähren von Beteiligungsmöglichkeiten angewiesen (Sturzenhecker 2013, S. 521). Inwiefern Perspektiven und Anliegen junger Menschen in Entscheidungsprozesse tatsächlich miteinfließen, bleibt bisher in der Regel wenig transparent (Schweda et al. 2020). Da eine glaubwürdige Beteiligung, in Abgrenzung zu einer Scheinbeteiligung, mit der Haltung und der Bereitschaft einhergeht, in politischen Beratungsprozessen Entscheidungsmacht zu teilen, ist Beteiligung stets auch mit Fragen von Macht und ungleichen Machtverhältnissen verbunden (von Schwanenflügel und Schwerthelm 2021, S. 988). Widerstände wie die Angst vor Machtverlust oder vor Veränderungen oder der Wunsch, eigene Privilegien und Machtpositionen beizubehalten, verhindern, junge Menschen in die Entscheidungsmacht über Beratungsgegenstände und -vorschläge miteinzubeziehen. Wenn das Recht auf Beteiligung im Sinne eines paternalistischen Entgegenkommens der Erwachsenen an die junge Generation gelebt wird, werden junge Menschen nicht als Rechtsträger_innen wahrgenommen und behandelt. Dadurch findet Beteiligung in politischen Beratungsprozessen vielmehr auf der Ebene des Gehörtwerdens statt (Knauer und Sturzenhecker 2005, S. 68). Power Sharing schließt ein, dass auch junge Menschen Themen selbst auf die Agenda setzen können. Eine glaubwürdige, generationengerechte Beteiligung junger Menschen in der Politikberatung bedeutet für erwachsene Akteure, die im generationalen Gefüge eine privilegierte und machtvolle Position innehaben, diese zu reflektieren, zu hinterfragen sowie zu teilen und sich auf damit verbundene Aushandlungsprozesse einzulassen.

Sowohl altersgemischte Gremien als auch Parallelgremien aus jungen Menschen wie ein Jugendbeirat laufen Gefahr, dass sie Kinder- und Jugendbeteiligung kuratieren, wenn die tatsächliche Entscheidungsmacht letztendlich doch ausschließlich bei Erwachsenen liegt. Daher sind in der Zusammenarbeit mit jungen Menschen beständige und ehrliche Kommunikation sowie Modi zum Abbau und zur Regulierung der Machtasymmetrie in den jeweiligen Strukturen fest zu verankern.

## Beteiligung junger Menschen im Kontext von sozialen Ungleichheiten und gesellschaftlicher Diversität

Je diverser eine Gesellschaft ist, desto wichtiger ist es für eine Demokratie, Entscheidungen mit den heterogenen Gesellschaftsmitgliedern rückzukoppeln. Angesichts ungleicher Ressourcenausstattung, negativer Zuschreibungen und daraus resultierenden unterschiedlichen Zugangschancen, spielt der Zugang zu Beteiligungsangeboten eine zentrale Rolle (Stange et al. 2021, S. 10). Viele Kinder, Jugendliche und junge Erwachsene können ihre Anliegen und Perspektiven aufgrund (struktureller) Barrieren, Ausschlüsse und unsicherer Lebensplanung nicht in politische Aushandlungsprozesse miteinbringen. Da (junge) Menschen unterschiedlich von sozialen Ungleichheiten betroffen sind, fallen auch ihre Handlungsspielräume und Einflussmöglichkeiten verschieden aus (BJK 2019, S. 11). Die Möglichkeit junger Menschen, sich an politischen Beratungsprozessen zu beteiligen, steht unter anderem in Zusammenhang mit Armutserfahrungen, Behinderung oder der Staatsbürgerschaft. Fehlende Zugänge aufgrund sozialer Ungleichheiten und strukturellen Diskriminierungen wirken einer breiten Beteiligung entgegen (Kutscher 2007; von Schwanenflügel 2015).

Soziale und ökonomische Benachteiligungen und Ausschlüsse wirken sich auf Beteiligungsprozesse aus, die wiederum soziale Ungleichheiten reproduzieren können. Es besteht die Gefahr, dass eine sozioökonomische Spaltung in der Gesellschaft zu einer partizipativen Spaltung führt (Kersting und Jähn 2021). Die Entwicklung niedrigschwelliger Zugänge und Zugangswege sowie einer diversitätsorientierten Auswahl junger Menschen für Gremien tragen dazu bei, dass sich auch junge Menschen beteiligen können, die nicht ohnehin bereits politisch engagiert sind (Knauer und Sturzenhecker 2005). Aus dem Bewusstsein, dass eine divergente Ressourcenausstattung die Beteiligungschancen beeinflusst und aus dem Anspruch heraus, Beteiligung in der Politikberatung so offen wie möglich zu gestalten, folgt die Notwendigkeit, Zugänge und die Ansprache junger Menschen niedrigschwellig und inklusiv zu gestalten sowie in Beteiligungsvorhaben die Diversität, die unterschiedlichen Erfahrungen und Lebenslagen junger Menschen zu berücksichtigen. Verschiedene Ansätze wie offene Strukturen, eine gezielte Ansprache, die Bereitstellung von ausreichend Personalmitteln, die Rücksichtnahme auf individuelle Bedarfe, professionelle Begleitung sowie die Gewährleistung barriere- und kostenfreier Räume können eine inklusive Beteiligung junger Menschen in der Politikberatung unterstützen (Kersting und Jähn 2021, S. 22). Beim Aufbau von Beteiligungsformaten mit repräsentativer Struktur wie zum Beispiel Beiräte oder Gremien, die mit jungen Menschen besetzt werden, kommt der ungleichheitssensiblen Auswahl und Begleitung der Mitglieder eine hohe Bedeutung zu.

## Verknüpfung von politischer Bildung und politischer Beteiligung

Die Beteiligung junger Menschen in der Politikberatung setzt voraus, dass junge Menschen Informationen über ihre Rechte, über mögliche Zugänge und Wege erhalten, wie sie sich einbringen können. Bisher werden junge Menschen noch nicht alterskonform über ihre Rechte informiert (Heyer et al. 2021; Stange und Lührs 2016). Der 16. Kinder- und Jugendbericht rückt die hohe Bedeutung von politischer/demokratischer Bildung in Verbindung mit politischer Beteiligung in den Fokus: Politische Bildung und politische Beteiligung stehen in einem engen Wechselverhältnis zueinander (Deutscher Bundestag 2020). Einerseits motiviert Beteiligung im politischen Kontext zur politischen Bildung und ist eine notwendige, aber noch nicht hinreichende Voraussetzung für Bildungsprozesse, anderseits erweitert politische Bildung das Repertoire möglichen politischen Handelns. Zur Anregung einer kritischen Analyse‑, Urteils- und Handlungsfähigkeit sind Beteiligungserfahrungen in Lernmöglichkeiten einzubetten (Deutscher Bundestag, S. 71, 80). Hierfür bedarf es der (Weiter)Entwicklung inklusiver Kommunikationswege, Informationen und Arbeitsweisen. Ziel ist es, junge Menschen dazu befähigen, sich selbst (politisch) zu positionieren, gesellschaftliche Prozesse zu verstehen und Politik mitzugestalten.

In diesem Zusammenhang zeigt die Shell Jugendstudie zu berücksichtigende soziale Unterschiede auf, die einen zentralen Auftrag der politischen Bildung implizieren: Je höher die Bildungsposition, desto höher das politische Interesse (Albert et al. 2019). Wenn politische Bildung ihre Angebote heterogener ausrichtet, die Anerkennung von diversen Lebenswelten und Kompetenzen einschließt sowie von einem tatsächlichen Abbau sozialer Ungleichheiten begleitet wird, kann sie zu einer Ermächtigung beitragen, die sich darin äußert, dass sich mehr (junge) Menschen berechtigt fühlen, sich politisch zu äußern und zu argumentieren (Bourdieu 1982, S. 639). Da die Prägekraft von sozialer Ungleichheit in Deutschland noch immer die biografische Verwirklichung und damit für die Lebensqualität und -perspektiven von Kindern und Jugendlichen zentral bestimmt (Novkovic 2021, S. 528), erfordert die politische Bildung ebenso wie die Beteiligung junger Menschen entsprechend inklusive Angebote, Räume, Formate und Zugänge, die auch benachteiligte Kinder, Jugendliche und junge Erwachsene erreichen. Gerade vor dem Hintergrund der Diagnosen über Demokratieentleerung und Desintegrationsdynamiken (Heitmeyer 2018) sowie Bemühungen daraus folgende gesellschaftliche Polarisierungen entgegenzuwirken, ist der Verknüpfung von politischer/demokratischer Bildung und Beteiligung (junger Menschen) eine hohe Relevanz beizumessen.

## Das Ringen um nachhaltige Beteiligung

Es gibt keinen Fahrplan oder keine allumfassende Schablone für Kinder- und Jugendbeteiligung. Formate und Methoden orientieren sich bestenfalls an jeweilige Alters- und Zielgruppen sowie Strukturen und Themen. Gleichwohl ist die Qualitätssicherung bei der Beteiligung junger Menschen vor dem Hintergrund fehlender Standards insbesondere auf Landes- und Bundeseben eine zentrale Entwicklungsaufgabe. Resümierend lassen sich einige allgemeine Anforderungen und Gelingensbedingungen für eine nachhaltige Beteiligung hervorheben:

- Eine selbstreflexive Haltung, die junge Menschen als Expert_innen für die sie betreffenden Themen anerkennt, korrespondiert mit einem transparenten und ergebnisoffenen Beratungsprozess.
- Die Bereitschaft, Macht abzugeben und mit jungen Menschen zusammen Beteiligungsformate für den jeweils spezifischen Kontext zu entwickeln, ermöglicht jungen Menschen, tatsächlich Einfluss zu nehmen und macht die Wertschätzung einer ernsthaften Beteiligung der jungen Generation sichtbar.
- Die Gestaltung niedrigschwelliger diversitätsbewusster Zugänge, Auswahlprozesse und Arbeitsweisen, die gezielt benachteiligte junge Menschen erreicht, wirkt sozialen Ungleichheiten entgegen.
- Erst mit ausreichenden Ressourcen ist es möglich, Beteiligung strukturell zu verankern. Ressourcen umfassen dabei nicht nur die materielle und personelle Ausstattung, sondern auch eine entsprechende Struktur, die für eine langfristig abgesicherte Beteiligung zentral ist. Dazu gehören auch Mittel und die Infrastruktur für begleitende Qualifizierungsangebote für alle beteiligten Akteure sowie für ausreichend Feedback und eine gemeinsame Evaluation, um Qualitätsstandards weiterentwickeln zu können.
- Die Bereitstellung altersgerechter sowie inklusiver Informationen bedeutet, die Verknüpfung von politischer Beteiligung und politischer Bildung zu stärken. Wenn Beteiligungsprozesse und Bildungsprozessen miteinander einhergehen, regen sie sich gegenseitig an und festigen die Demokratie.
- Ein transparentes, ebenenübergreifendes Monitoringsystem überprüft, inwiefern eingebrachte Anliegen und Vorschläge aufgenommen, getroffene Entscheidungen langfristig eingehalten sowie Beratungsergebnisse fortgeschrieben werden.

Eine strukturell verankerte Beteiligung junger Menschen kostet Ressourcen, verläuft nicht geradlinig und geht, wie generell eine demokratische Politik, mit Konflikten einher. Für eine Demokratie ist eine starke zivilgesellschaftliche Beteiligungsstruktur der jungen Generation, die teilweise noch nicht durch Wahlen ihre Stimme abgeben oder politische Ämter übernehmen kann, besonders bedeutsam. Die Mitwirkung von Vielen und (damit einhergehende) gesellschaftliche Veränderungen führen zu Reibungen, die Treibstoff für Fortschritt sein können (El-Mafaalani 2020, S. 152). Damit eine Kultur der Beteiligung junger Menschen selbstverständlich werden soll, braucht es Mut, neue Beteiligungsformate mit jungen Menschen gemeinsam auszuprobieren sowie eine konstruktive Diskussions- und Fehlerkultur, die zu einer Qualitätssicherung von Beteiligungsprojekten beitragen. Eine ungleichheitssensible und generationengerechte Überarbeitung oder Neuauflage der ressortübergreifenden Jugendstrategie der Bundesregierung wäre ein Zeichen in die Richtung einer nachhaltigen und glaubwürdigen Beteiligung junger Menschen in der Politikberatung auf Bundesebene.
